# Institutional Notes and News

**Published:** 1910-01-01

**Authors:** 


### Institutional Notes and News.

**A Note from Sheffield.**

At a meeting of the Sheffield Hospital Sunday Committee it was unanimously decided to hold the next Hospital Sunday collection on January 30. Mr. John Marshall, who presided, stated that it had been arranged that the financial year of the Sheffield Royal Infirmary should end on December 31 instead of June 30 as hitherto. They had fallen into line with most of the medical charities in the country, particularly with London. He did not know whether they would be able to participate in the Prince of Wales's grant, but one of the conditions which hospitals in receipt of that benefit had to comply with was to end their financial year on December 31.

**Worcester General Infirmary.**

At the monthly meeting of the executive committee a statement of the present financial position was made by the chairman, in view of the proposed buildings for the laundry and children's ward. He showed that, although the funded property amounted to about £52,395, most of it was either earmarked or Corporation stock, which is unsaleable, and there remains only about £2,000 of marketable stock. A plan of the proposed alteration to the laundry was presented, which would cost about £600 and not provide steam. The matter was debated, and postponed for the purpose of ascertaining the estimated cost of working by steam and what saving would be effected thereby. With regard to the proposed children's hospital, the senior physician reported that the medical staff recommended, instead of a separate building, that the north wing of the infirmary be lengthened to correspond to the south wing, and that one of the wards on the south side should be set apart for the children's ward and provided with a covered balcony. Emphasis was laid on the present inadequate and insufficient accommodation for children, cases having sometimes to wait for months before they can be admitted. It was estimated that the cost of this building should not exceed £4,000. The Mayor supported the medical staff in their plea for the urgency of this extension.

**Practical Help.**

At the half-yearly meeting of the Yarmouth Hospital Committee the question of the financial position of the institution was discussed. There will be a deficit of nearly £1,600 if the new additions proposed are to be carried out. The Hon. Secretary said the position was serious, as their receipts were below their ordinary expenditure. When they opened the new ward their expenditure would be increased by £300 or £400 a year, so that further annual subscriptions were needed not only to meet the present deficiency, but to enable them to cover the future additional expenditure. They would be out of pocket by £1,500 when they had completed the new ward, and it meant the loss of interest at 3½ per cent., which was £55 per annum, for their ordinary income. In the course of the discussion one of the governors said that a great deal might be done by organised assistance if the large firms would only help. A Scotchman who came there the other day said that in the North they paid 6d. per week for the infirmary. When he engaged a Scotchman and paid him at the end of the week he put a penny or twopence on the counter at the week-end for the infirmary. This plan was also followed at Norwich. All they had to do was to get the men to agree to it, and then this penny was paid week by week. He felt sure the working men would agree to it. The accounts were finally passed.

**Ladywell Workhouse.**

At the last meeting of the Bermondsey Guardians the Ladywell Workhouse Committee reported that, after carefully considering and discussing in detail reports of the Local Government Inspector and the observations of the medical officer of the Ladywell Workhouse thereon, and especially parts relating to diets, extras, and stimulants, it had come to the conclusion to entirely disagree with the proceedings of the medical officer of that workhouse, considering the same to be generally unsatisfactory. A recommendation was submitted, but after a long discussion upon procedure the further consideration of the matter was adjourned.

**The Ladies' Guild of the Royal Free.**

Princess Marie-Louise of Schleswig-Holstein opened the annual exhibition of Christmas presents for patients provided by the Ladies' Association of the Royal Free Hospital, and of the garments from the Princess Marie-Louise Guild of Work, of both of which organisations the Princess is the president. Her Highness was received by the ladies who are vice-presidents of the Guild and by Miss Cox-Davies, the matron, and spent some time in an inspection of the articles displayed. The Guild has supplied nearly nine hundred garments for the use of patients while they are in the institution, and the Association sends 350 articles of clothing and other things for distribution as Christmas gifts to inmates from a district which Mr. Charles Booth described as having 61 per cent. of its residents belonging to the class which is living in absolute poverty. The Guild has also made a money grant of £30, and the Association has given £50 for the support of a bed, so that the good work done by the ladies who are associated in these efforts is manifest.

**A Field Day at York.**

An interesting ceremony took place at York County Hospital last week, when the High Sheriff of Yorkshire (Mr. G. W. Lloyd, J.P.), as a representative of the county, formally opened the extensions to the Nurses' Home connected with the hospital. It had been intended that the Dean of York should conduct a service in dedication of the cot in the children's ward which has been endowed by public subscription in memory of the late Canon Fleming, but owing to a pronounced case of scarlet fever having occurred in that ward this ceremony had to be indefinitely postponed. The extensions consist of eighteen additional bedrooms, with bathroom and lavatory conveniences, thus increasing the total accommodation to thirty-eight bedrooms, sitting-rooms, seven bathrooms, and lavatories. The total cost of the whole building is about £6,600, and of furnishing about £800. Of this gross total of about £7,400 only £52 has been provided by subscription; the remainder will have to be provided from other sources, chiefly from capital, and probably from the sale of investments. The building of the extensions has cost £2,340, and the furnishing about £250, the former being over £90 below the estimate, so that a substantial saving has thus been effected. There are about forty nurses on the general staff, and the extra accommodation will be greatly appreciated. The rooms are light and airy, the dimensions of the majority of the bedrooms being a little over ten feet by six feet, while the sitting-rooms are most inviting retreats. There has been no expense wasted upon anything of an ornamental or really unnecessary character, though the reasonable comforts of the staff have been considerately kept in view.

